# What Drives Portuguese Women to Be Physically Active? Associations between Motives and Well-Being Indicators

**DOI:** 10.3390/ijerph20043352

**Published:** 2023-02-14

**Authors:** Alicia Silva, Raul Antunes, Diogo Monteiro, Miguel Jacinto, Rui Matos, Filipe Rodrigues

**Affiliations:** 1 ESECS—Polytechnic of Leiria, 2411-901 Leiria, Portugal; 2 Life Quality Research Centre (CIEQV), 2040-413 Leiria, Portugal; 3 Center for Innovative Care and Health Technology (ciTechCare), Polytechnic of Leiria, 2411-901 Leiria, Portugal; 4 Research Center in Sport Sciences, Health Sciences and Human Development (CIDESD), 5001-801 Vila Real, Portugal; 5 Faculty of Sport Sciences and Physical Education, University of Coimbra, 3040-248 Coimbra, Portugal

**Keywords:** exercise, health, self-determination theory, motives

## Abstract

Motives and self-esteem play crucial roles in shaping personal behavior and emotions and have been shown to impact well-being. However, the association between these constructs has been overlooked in women who seem to be more externally driven to engage in exercise. The present study was carried out with the objective of analyzing the associations between motives for physical exercise, positive and negative activations, and self-esteem of Portuguese women exercising at gyms and fitness centers. The sample consists of 206 women aged between 16 and 68 years old (M = 35.77; SD = 11.47). Participants answered a short sociodemographic questionnaire, the Goal Content for Exercise Questionnaire, the Positive and Negative Affect Schedule, and the Rosenberg Self-esteem Scale. The results showed that the health motive had the highest predictive value (β = 0.24; *p* < 0.01) on self-esteem and demonstrated a positive and significant correlation with positive activation and self-esteem (*p* < 0.01). On the other hand, the social recognition motive had the lowest predictive value on self-esteem (β = −0.04; *p* > 0.05) and demonstrated a non-significant correlation with positive activation and self-esteem (*p* > 0.05). Looking at the coefficients in the hierarchical regression model, it can be seen that the health motive and positive activation were positively and significantly correlated with self-esteem. This study points to the need to raise awareness about the motives of exercise related to the physical and mental health of Portuguese women. Portuguese women that exercise for health motives display greater perceived self-esteem which is an indication of a greater sense of well-being. While the results are limited to Portuguese women, exercise physiologists assessing exercise motives could provide information on how to prescribe exercise as a means to increase self-esteem, considering the positive activation resulting from this behavior.

## 1. Introduction

Scientific data show significant health advantages from regular physical activity and exercise, which plays a vital role in the prevention and treatment of a wide range of chronic diseases, health issues, and risk factors [1]. Women are less active than men in most countries [1,2,3]. In the European Union, for example, physical inactivity appears to be more prevalent in women (49%) than in males (40%). In Portugal, 80% of women and 75% of men say they never or rarely exercise [2].

Previous research, both in men and women, reveal various adaptations to regular exercise, highlighting some substantial differences in terms of growth in muscle strength, range of motion, and risk of injury [4]. Related to women specifically, exercise appears to be associated with a lesser proclivity to gain excessive weight during pregnancy, resulting in a lower risk of developing gestational diabetes and postpartum depression [5]. In addition to these effects, regular physical exercise appears to have an important role in postmenopausal women, whose hormonal changes put them at a higher risk of severe bone density loss, osteopenia, developing osteoporosis, severe osteoarthritis, and other musculoskeletal conditions and diseases [6].

### 1.1. Motives for Exercise Participation

The involvement in exercise by Portuguese women in the fitness sector is decreasing [7], where 80% report not engaging in this behavior. Thus, it is critical to focus efforts on understanding the motives and motivations that can lead to the adoption and maintenance of regular exercise [8]. Dominating socio-cognitive frameworks have been used to investigate the motives driving humans to engage in sufficient physical activity. Empirical studies [9,10,11] have demonstrated encouraging results in the exercise context, demonstrating that autonomous motivation, perceived benefits, social support, and intentions are major predictors of exercise adherence. Related to these motivational determinants, motives are crucial in the effort and achievement of any type of behavior. Motives are defined as the process of initiating and maintaining goal-directed behavior. As a result, motive can be defined as a behavioral energy that determines whether or not a behavior is executed. Self-determination theory, developed by Deci and Ryan [12] and later investigated in the exercise domain by Rodrigues et al. [13] and Teixeira et al. [14], posits that individuals have a natural tendency to respond to a given behavior following their motivational state. This socio-cognitive theory is concerned with causes, such as reasons for engaging in exercise and consequences of self-determined behavior.

It should be noted that reasons and motivation are two independent concepts [12], even though self-determination theory connects them as “drivers” towards a given behavior. A motive is “what” a person expects to gain as a result of engaging in a particular behavior (e.g., “I exercise to enhance my health”). On the other hand, motivation is “how” an individual will put effort towards the behavior (e.g., “I exercise because I identify myself as a healthy person”). Grounded in self-determination assumptions, Ryan and Deci [12] distinguish motives between intrinsic and extrinsic reasons. Intrinsic motives are those that bring enjoyment at work or in the environment in which they engage, as well as those that try to develop personal interests, values, and potential while being naturally joyful to perform [13]. Intrinsic reasons include health, skill advancement, fun, pleasure, and vitality. These motives are internal, which is why personal improvement is actively pursued [15]. Intrinsic motives differ from intrinsic and autonomous motivation [16]. An individual, for example, may act altruistically, with an internal goal, simply to please a family member or friend, an extrinsic behavioral regulation. Thus, despite their association with intrinsic and autonomous motivation, intrinsic motives can also direct motivation towards extrinsic factors [17]. On the other hand, extrinsic reasons are external, oriented “outside” of the individual, and hence sought after by external contingencies [18]. Money, fame, and social recognition are examples of extrinsic motives that urge individuals to direct their motivation through extrinsic regulations (e.g., external regulation and introjected regulation). These goals frequently block the fulfillment of autonomy, competence, and relationships, which undermines the individual’s well-being and personal growth [12]. According to studies on exercise motives, men and women have different motivational drivers for this behavior [19]. Several studies show that intrinsic reasons (e.g., physical fitness, competence, and challenge) predominate in men, while extrinsic motives (e.g., weight control and appearance) predominate in females [15,20,21].

### 1.2. Associations between Motives, Self-Esteem, and Affect

Previous studies have shown that having high intrinsic aspirations relative to extrinsic reasons was associated with significantly greater well-being [12]. Previous research has also shown that pursuing intrinsic, rather than extrinsic, life goals have implications not only for personal well-being but also for personal social functioning, measured by a greater cognitive evaluation of the self [22]. In short, prior research on intrinsic versus extrinsic motives suggests that the content of goals is paramount. However, research in diverse life domains is still limited. Self-esteem has been studied since it is a key component of happiness and well-being [23]. Self-esteem is a fluid term that arises from the interaction of factors related to the self, the individual, and his perspective of himself. It is associated with evaluative and emotional parts of the self-concept, as a result of the assessment that the individual generates about personal talents and performance, while not forgetting the affective component associated with personal identity [24].

There is significant evidence that exercise can influence perceptions of personal well-being in a favorable way, increasing self-esteem and, as a result, changing sedentary into more active behaviors [25,26]. Existing studies [9,25,27], found a link between increased physical activity and higher self-esteem. Other studies demonstrate that physical activity can improve mood and happiness, as well as reduce tension induced by stressful events, especially shortly after exercise, while also increasing self-esteem, self-concept, and self-confidence [28,29,30]. Individuals with a higher sense of self-worth may feel this way as a result of an increase in positive activations and a decrease in negative activations. In contrast, several researchers [31,32] have demonstrated that people with high levels of negative activations are more vulnerable to risks to their self-esteem compared to those reporting higher levels of positive activations.

The affective component is also very important in the concept of subjective well-being. Diener [33] defines well-being as a cognitive (satisfaction with life) and affective (i.e., positive and negative activations) assessment that individuals make about their own lives, which can be judgments such as satisfaction with life, but also affect-based assessments [34]. Affect can be considered as hedonic motivation that interacts with environmental stimuli. Positive activations are factors of a pleasurable experience (e.g., happiness, joy, among others), while negative activations are associated with negative factors that are characterized by an unpleasant experience (e.g., loneliness, sadness, guilt, among others), as described by Crawford and Henry [35]. As a result, having more positive activations and fewer negative activations may result in higher levels of well-being [36] specifically self-esteem [9]. Individuals who exercise regularly have higher levels of well-being levels than those who do not practice [37,38]. Individuals with greater levels of well-being are more physically active, according to many studies [39,40,41,42].

### 1.3. Current Study

While the relationship between positive and negative activations and well-being indicators appears conceptually sound, the studies cited have been undertaken in non-physical-activity contexts [31,43]. To the best of our knowledge, little research has looked at the relationship between physical exercise, affective activations, and how people see themselves in the exercise context [9]. Examining the relationship between exercise motives and the affective activation to this behavior may provide additional insight into strategies for promoting regular exercise. The fundamental rationale for looking at the positive consequences of the affective activation to regular exercise is that a lack of physical activity may lead to a low self-esteem [9,10,11,31] and a higher risk of mental distress indicators, such as depressive symptoms [44]. Researchers have also postulated pathways via which physical exercise and affective motivation could contribute to enhanced mental health [45], implying that higher levels of positive activations and lower levels of negative activations are related to higher levels of adaptive outcomes [12].

The present study was carried out with the objective of analyzing the associations between motives for physical exercise, positive and negative activations, and self-esteem of Portuguese women exercising at gyms and fitness centers. Based on past research, we hypothesized that intrinsic motives would be positively and significantly associated with positive activations and self-esteem [9,12,46]. We also hypothesized that extrinsic motives would be negatively and significantly associated with positive activations and self-esteem [9,12,46]. Looking at exercise motives, we speculate that intrinsic motives would have the most significant associations with self-esteem compared to extrinsic motives [9,12,46].

## 2. Materials and Methods

### 2.1. Participants

The a priori sampling calculator for the hierarchical multiple regression analysis [47] was used to calculate the minimum sample size required for this study to be valid and reliable. The following inputs were used: anticipated effect size for set B = 0.2; desired statistical power = 0.95; number of predictors in set A = 5; number of predictors in set B = 2; and probability level = 0.05. The results suggest that the minimum number of participants is 85 for the results to be valid and reliable.

In this study, 206 women aged 18 to 68 years (M = 35.77; SD = 11.47) were included for analysis. Concerning body mass index (M = 23.02; SD = 3.59), participants self-reported height and weight, indicating to have normal weight (*n* = 140; 68.00%), overweight (*n* = 55; 26.70%), or obese (*n* = 11; 5.30%). For inclusion, we considered those who met the following inclusion criteria: (i) aged 18 years or older; (ii) provide informed consent to participate; and (iii) be an active gym or fitness center member for at least 6 months. These criteria are based on an exercise maintenance curve in which individuals have approximately a 50% ratio of continuing or dropping out from exercising [9,11].

### 2.2. Procedures

The data were collected per the Helsinki Declaration [48], and the data collection procedure was approved by the Ethics Committee of the Polytechnic of Leiria (reference number: CE/IPLEIRIA/35/2021). The current study design was cross-sectional, and an online questionnaire using the Google Forms platform was created and later shared on different social networks. The authors clearly stated in the disclosure section that only women were considered for this study. Objectives for this study were explained to all potential participants and signed informed consent was obtained individually by checking the consent box. Participants completed measures using self-administered instruments. All Portuguese women completed the questionnaires in full due to how the google forms was created and thus data from all participants were considered. The mean time to complete questionnaires was approximately 10 min. Data collection took place between the 7 February and the 30 October 2022.

### 2.3. Instruments

The Goal Content Exercise Questionnaire Portuguese version [49] was used to evaluate intrinsic and extrinsic goals that individuals can pursue in the exercise context. This measure comprises 20 items evaluated on a 7-point Likert scale ranging from 1 (“Totally Disagree”) to 7 (“Totally Agree”). Subsequently, scores are grouped into five factors (each with four items), namely (a) health; (b) skills development; (c) social affiliation; (d) image; and (e) social recognition. This scale has shown to be a valid and reliable measure of intrinsic and extrinsic motives, suited for use with a wide range of age groups and empirical applications in the exercise context [16].

The Rosenberg Self-esteem Scale Portuguese Version [50] to assess self-esteem was utilized. While the original version has 10 items, we only evaluated five items that were positively coded (item example, “I believe I have several good traits”) because reverse-coded items can contaminate results [51]. Participants in the present study used a 4-point Likert scale to indicate how strongly they agreed or disagreed with each statement, ranging from 1 (“strongly agree”) to 4 (“strongly disagree”). This scale is the most extensively used measure of self-esteem for research purposes, and it has demonstrated adequate model fit [52], as well as acceptable internal consistency score [53].

The Satisfaction with Life Scale Portuguese version [37] was used to measure satisfaction with life. This 5-item scale is designed to measure global cognitive judgments of personal life satisfaction, and study participants responded to each item using a 7-point scale ranging from 1 (“totally disagree”) to 7 (“strongly agree”). This scale has been shown to be a valid and reliable measure of life satisfaction, suited for use with a wide range of age groups and empirical applications [54].

### 2.4. Statistical Analysis

Using the IBM SPSS STATISTICS version 25.0 (IBM Corp., Armonk, NY, USA) software, descriptive statistics, such as means and standard deviations, as well as bivariate correlations between all variables under consideration were generated. To calculate the statistical significance of a deviation from the normal distribution, the skewness and kurtosis estimations were divided by their standard errors to yield the z score. A z score less than |1.96| indicated a normal distribution. For the referred analyses, a significance value ≤ 0.05 was assumed to reject the null hypothesis [55].

Hierarchical multiple regression analyses were conducted to test the proposed associations. Before performing a regression analysis, tolerance test and variance inflation factor (VIF) scores were analyzed to test for possible multicollinearity issues [56]. The tolerance of independent variables should be greater than 0.1 and VIF score should be lesser than 5 for there to be no multicollinearity issue. The Durbin–Watson statistic test for autocorrelation was also calculated, assuming an acceptable range of 1.50–2.50 [57]. Self-esteem was imputed as the dependent variable. We used the stepwise procedure, as we intended to add variables following theoretical assumptions. In model 1, we imputed intrinsic and extrinsic motives. In model 2, positive activation was imputed, and in model 3, we imputed negative activations. Models were compared using the R^2^ and changes were analyzed using the significance level at *p* ≤ 0.05 to reject the null hypothesis [55].

## 3. Results

Descriptive statistics and bivariate correlations are shown in Table 1. The mean score for health was greater than all other exercise motives. Skewness and kurtosis values were below cutoffs indicating a normal distribution. Several significant bivariate correlations emerged as expected, namely (a) intrinsic motives were positively associated with positive activations; (b) extrinsic motives were positively associated with negative activations; (c) intrinsic motives were positively associated with self-esteem; and (d) extrinsic motives were positively associated with self-esteem.

The results of the hierarchical multiple regression are presented in Table 2. The tolerance values ranged from 0.52 to 0.89. In addition, the VIF values ranged from 2.17 to 3.12. Therefore, there were no multicollinearity issues in this analysis. The Durbin–Watson test indicated a score of 1.91, indicating an acceptable score close to zero autocorrelation. We checked the significance (*p*-value) of the model to examine whether the model is significantly different from a null hypothesis. We checked the R^2^ value to see how much of the variance of self-esteem was explained by the model. To identify which variable contributed most to the model, we checked the standardized coefficients and significance of the independent variables. In the hierarchical multiple regression analysis, we compared the models as variables were added (changes in R^2^). In general, model 1 containing only exercise motives explained 9% of the variance of self-esteem. In turn, model 2 including positive activations explained 37% of the variance of self-esteem, and model 3 including negative activations explained 44% of the variance of self-esteem. Based on the F coefficient, the results indicate that the third model is the most parsimonious compared to the previous models to have predictive abilities. Looking at the coefficients, it can be seen that in model 3, health motive and positive activation were positively and significantly correlated with self-esteem. On the other hand, negative activation was negatively and significantly correlated with self-esteem.

## 4. Discussion

The present study was carried out with the objective of analyzing the associations between motives for physical exercise, positive and negative activations, and self-esteem of Portuguese women exercising at gyms and fitness centers. According to the current findings, the mean score for the health motive was higher than other exercise motives in Portuguese women. Several strong bivariate correlations appeared, as predicted by theory, including a significant connection between intrinsic motives and self-esteem. Furthermore, extrinsic motives and negative activations were inversely associated with self-esteem.

The health motive showed a positive and significant correlation with self-esteem. This result supports existing literature [58,59], which suggest that some intrinsic reasons can boost self-esteem by allowing people to engage in activities that they enjoy, giving them a sense of pleasure and personal accomplishment. Intrinsic motive is the desire to perform something for the sake of its intrinsic satisfaction rather than for any external benefit. Health and skill development were both significantly correlated with positive activation. It is theoretically expected that intrinsic motive can lead to increased enjoyment and engagement in tasks that are perceived as pleasurable, as well as a greater mastery of skills and improved performance [60]. Intrinsic motive is also linked with higher levels of self-confidence, self-efficacy, and a greater sense of autonomy [9]. Women engaging in regular exercise can lead to greater perceived intrinsic motivation as it can help foster a greater sense of connection and purpose which is associated with the experience of positive activations.

Extrinsic motives were positively associated with negative activation, as shown in Table 1. These results are in accordance with previous studies [9,16], showing that extrinsic reasons increase the likelihood of experiencing negative consequences related to exercise. Extrinsic motives can lead to the over justification effect, where the individual starts to rely on the reward and loses their intrinsic motivation. This can result in a decrease in the perceived pleasure of an activity or task. Additionally, extrinsic motivation can lead to an unhealthy dependency on rewards, which can have negative consequences on well-being.

The results showed that self-esteem was positively correlated with positive activation, and negatively with negative activation. The result supports the argument that affective states are particularly important in defining levels of self-esteem [31,61,62]. Similarly, Swickert et al. [63] found no support for the role of negative activation in its relationship with self-esteem, suggesting that positive and negative affect exert different effects in determining self-esteem. Exercise, in turn, has shown a positive association with self-esteem, with several studies suggesting that exercise provides an important contribution to improving levels of self-esteem [29,64,65,66,67]. Elavsky and colleagues [68], in a 2-year experimental study, concluded that women could improve their perception of their physical condition and, consequently, increase self-efficacy, and self-esteem by engaging in regular exercise.

Results showed that the health motive and positive activation were positively associated with self-esteem. As described, self-esteem seems to be related to affective responses, so people with higher levels of self-esteem may feel this due to an increase in positive activation [22,31,69]. Intrinsic motives, when connected with positive activations, offer a better understanding of self-esteem, particularly in women [70]. This should lead exercise physiologists not only to understand the reasons why women engage in regular exercise, but also creating conditions for them to sense positive activations [60].

### 4.1. Limitations and Suggestions for Future Studies

There are certain limitations to this study that must be noted. The first is that the sample is limited to Portuguese women who exercise in the context of gyms and health clubs. As a result, the findings cannot be generalized to other circumstances or people. The current study offers a preliminary look at the mechanisms that may translate exercise motives into a more positive and subjective experience of exercise, ultimately translating into self-esteem in Portuguese women. To better understand the mechanisms underlying this relationship, the replication and extension of current findings using additional potential mediators, such as basic psychological needs, behavioral regulation, and objectively measured outcomes (e.g., exercise participation using attendance records), are required. Related to limitations of the current study, participants were regular exercisers recruited from a convenience sample of health and fitness centers; we did not account for exercise intensity and duration, which appear to be associated with the affective response to exercise [71]. Future research should consider the essential problem of the timing of the measurement of affective activations [72,73]. The cross-sectional design used in this study should be investigated using longitudinal or prospective methods to test causal relationships in forthcoming studies. Another restriction is that only adaptive outcomes are considered. While the goal was to look into the relationship between exercise motive, affective activations, and self-esteem, future research should look into the predictive impact on maladaptive consequences (e.g., burnout, psychological distress, anxiety) in the exercise context. Finally, while this study did not measure the fitness activities exercisers engaged in, future studies could assess and investigate any differences between gym and health club activities, such as calmer (e.g., pilates) versus more enthusiastic and intense (e.g., crosstraining, pump) exercise types.

### 4.2. Implications for Pratice

According to the results that support the current investigation, it is acknowledged that simply following exercise programs without assessing motives may not be sufficient to sustain an active lifestyle and may, in many cases, have a negative outcome (e.g., early dropout). Furthermore, the practitioner-instructor relationship appears to be one of the primary variables defining this adherence [74]. According to the findings of this study, it is critical that exercise professionals understand how to promote supporting contexts for women’s needs, and that they adjust their intervention practices with considerations for promoting positive activations, facilitating the development of more autonomous forms of motivation, and thus increasing positive responses to exercise [14].

## 5. Conclusions

Exercise motives can help individuals to achieve a positive self-concept and promote psychological well-being. Furthermore, individuals with increased self-esteem are motivated to exercise as they feel capable of fulfilling the exercise regimes that satisfy their self-worth. Understanding which factors affect exercise participation will enable specialists to create exercise regimes tailored to individual needs, which will ultimately increase exercise engagement. The health motive is an important factor in our overall well-being. It is a powerful motivator for making changes in our lives and for achieving our goals. Strategies such as recognizing situations that affect self-esteem, challenging negative thinking, adjusting thoughts and beliefs, and taking steps to care for oneself can all help to boost self-esteem and improve overall mental and physical health in Portuguese women.

## Figures and Tables

**Table 1:** Descriptive statistics and correlations.

Variables	M	SD	S	K	1	2	3	4	5	6	7	8
1. Social Affiliation	3.54	1.52	0.27	−0.58	1	-	-	-				
2. Image	4.70	1.28	−0.28	−0.59	0.42 **	1	-	-				
3. Health	6.47	0.62	−1.26	0.97	0.23 **	0.29 **	1	-				
4. Social Recognition	2.36	1.38	1.14	0.78	0.61 **	0.51 **	0.12	1				
5. Skill Development	5.42	1.33	−1.02	0.88	0.39 **	0.29 **	0.43 **	0.25 **	1			
6. Positive Activation	3.79	0.65	−0.60	1.45	0.03	−0.08	0.16 *	0.13 *	0.13 *	1		
7. Negative Activation	2.34	0.88	0.82	0.71	0.18 **	0.24 **	0.03	0.23 **	0.07	−0.44 **	1	
8. Self-Esteem	3.98	0.67	−0.38	−0.62	0.03	−0.10	0.27 **	−0.02	0.10	0.58 **	−0.35 **	1

Notes: M = mean; SD = standard deviation; S = skewness; K = kurtosis; * = *p* < 0.05; ** = *p* < 0.01.

**Table 2:** Standardized beta coefficients and explained variance.

Model	*R* ^2^	Δ*R*^2^	F	β	t
**Model** 1	0.09	0.11 *	5.07	-	-
Social Affiliation	-	-	-	0.007	0.17
Image	-	-	-	−0.23 **	−2.84
Health	-	-	-	0.33 **	4.33
Social Recognition	-	-	-	0.06	0.62
Skill Development	-	-	-	−0.003	0.04
**Model** 2	0.37	0.27 *	20.77	-	-
Social Affiliation	-	-	-	0.08	1.06
Image	-	-	-	−0.09	1.23
Health	-	-	-	0.23 **	3.56
Social Recognition	-	-	-	−0.10	−1.37
Skill Development	-	-	-	−0.05	−0.79
Positive Activation	-	-	-	0.55 **	9.39
**Model** 3	0.44	0.06 *	22.85	-	-
Social Affiliation	-	-	-	0.08	1.12
Image	-	-	-	−0.07	−0.98
Health	-	-	-	0.24 **	3.89
Social Recognition	-	-	-	−0.04	−0.51
Skill Development	-	-	-	−0.05	−0.73
Positive Activation	-	-	-	0.44 **	7.38
Negative Activation	-	-	-	−0.28 **	−4.70

Notes: β = standardized coefficients; t = *t*-test; R^2^ = adjusted r square—explained variance; Δ = differences; F = changes in significance; * *p* < 0.01; ** *p* < 0.01.

## Data Availability

Not applicable.
